# Modifiers of the Genotype–Phenotype Map: Hsp90 and Beyond

**DOI:** 10.1371/journal.pbio.2001015

**Published:** 2016-11-10

**Authors:** Rachel Schell, Martin Mullis, Ian M. Ehrenreich

**Affiliations:** Molecular and Computational Biology Section, Department of Biological Sciences, University of Southern California, Los Angeles, California, United States of America

## Abstract

Disruption of certain genes alters the heritable phenotypic variation among individuals. Research on the chaperone Hsp90 has played a central role in determining the genetic basis of this phenomenon, which may be important to evolution and disease. Key studies have shown that Hsp90 perturbation modifies the effects of many genetic variants throughout the genome. These modifications collectively transform the genotype–phenotype map, often resulting in a net increase or decrease in heritable phenotypic variation. Here, we summarize some of the foundational work on Hsp90 that led to these insights, discuss a framework for interpreting this research that is centered upon the standard genetics concept of epistasis, and propose major questions that future studies in this area should address.

## Perturbation of Hsp90 Impacts Heritable Phenotypic Variation

How particular genes modulate the heritable phenotypic variation that segregates within populations is not fully understood. Work on the chaperone Hsp90 has generated some of the most provocative insights into this problem. In a landmark paper, Rutherford and Lindquist demonstrated that perturbation of Hsp90 reveals heritable phenotypic variation among genetically distinct *Drosophila* isolates [1]. Similar experiments in *Arabidopsis* [2] and yeast [3] subsequently showed that this effect generalizes across species.

**Fig 1:**
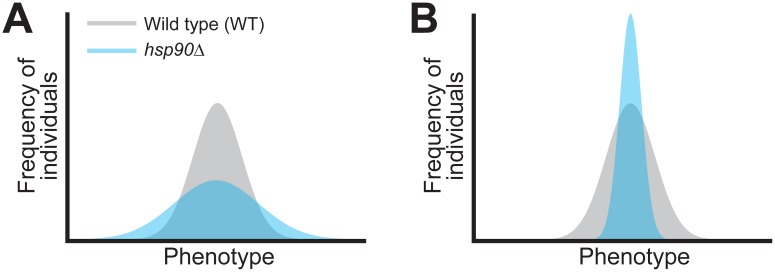
Effects of genetic buffers and potentiators on heritable phenotypic variation. In both **A** and **B**, the gray distributions indicate when a buffer or potentiator is functional, while the blue distributions show when a buffer or potentiator is perturbed. As shown in **A**, buffers suppress heritable phenotypic variation among individuals. If these buffers are compromised, heritable phenotypic variation increases. **B** illustrates how potentiators act in an opposite manner to buffers, with heritable phenotypic variation decreasing when potentiators are disrupted.

The initial interpretation of these results was that Hsp90 is a buffer that suppresses the effects of genetic variation on a global scale [1,2] (Box 1). Perturbation of Hsp90 was thus thought to uncover cryptic polymorphisms that do not typically show effects, thereby expanding the amount of heritable phenotypic variation in a population [1,2,4–7] (Fig 1A). Because of its role in buffering, Hsp90 began to be regarded as a capacitor that facilitates the accumulation of cryptic genetic variation [1–3,8–12].

**Box 1. Glossary of Relevant Terms**

**Capacitor**. A gene that facilitates the accumulation of cryptic genetic variation.

**Cryptic genetic variants**. Genetic variants that only show effects under atypical conditions, such as when specific genes are compromised or the environment markedly changes.

**Epistasis**. When the effect of one variant is influenced by one or more other variants.

**Genetic buffer**. A gene that suppresses the effects of one or more variants.

**Genotype–phenotype map**. The correspondence between genotypes and the phenotypes they produce.

**Global modifier**. A gene that acts as an epistatic modifier for many variants.

**Magnitude epistasis**. An epistatic interaction that involves a variant’s effect size changing while its effect sign remains the same.

**Modifier**. A gene or variant that influences the effect of another through an epistatic interaction.

**Mutation accumulation lines**. Strains that have accumulated new mutations during many generations of minimal selection.

**New mutations**. Recently arisen variants that have not yet been exposed to natural selection.

**Potentiator**. A gene that enables certain variants to exhibit effects.

**Sign epistasis**. An epistatic interaction that involves a variant’s effect changing in sign (i.e., from positive to negative or vice versa).

**Standing genetic variation**. Genetic variation that segregates within populations and may have been subject to natural selection.

The buffer and capacitor concepts were appealing because of Hsp90’s function as a chaperone for many key signaling proteins and transcriptional regulators [13,14]. However, as additional studies examined the influence of Hsp90 on genetic variation, a more complex picture emerged. Contrary to previous findings, instances were found in which perturbation of Hsp90 reduced heritable phenotypic variation [3,8] (Fig 1B). This suggested that Hsp90 does not always act as a genetic buffer but instead sometimes makes possible (or potentiates) the effects of genetic variation [3,8].

## Selection Complicates Inferences about Buffering and Potentiation by Hsp90

**Fig 2:**
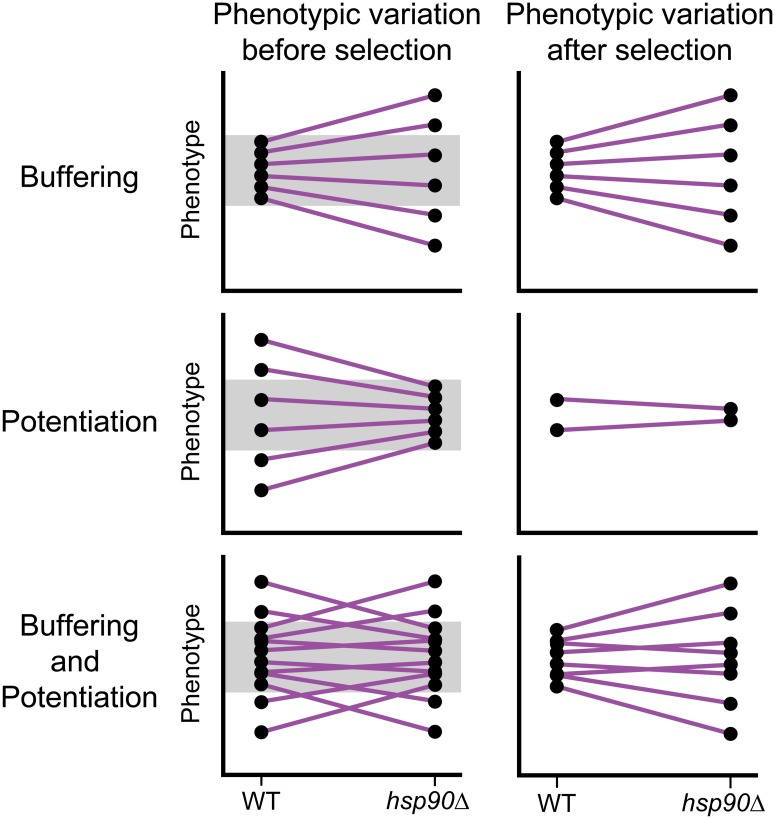
Inferences about the extent of buffering and potentiation are affected by natural selection. Here, we illustrate how selection may lead to misinterpretations regarding the extent to which Hsp90 acts as a buffer and a potentiator by disproportionately acting against Hsp90-potentiated heritable phenotypic variation. We present three scenarios: Hsp90 exclusively acts as a buffer, Hsp90 exclusively acts as a potentiator, and Hsp90 acts equally often as a buffer and a potentiator. In each case, we show the genotypes and heritable phenotypic variation that existed prior to selection as well as the genotypes and heritable phenotypic variation that remain after selection. Within a given Hsp90 state, black circles represent distinct genotypes. Purple lines indicate the change in an individual’s phenotype upon Hsp90 perturbation. Gray boxes show the range of phenotypes that are tolerated by selection under normal conditions in which Hsp90 is functional. In this figure, we only consider selection that acts to stabilize the population around a particular mean value.

The extent to which Hsp90 acts as a buffer or a potentiator has bearing on our expectations for how new mutations and standing genetic variants will affect heritable phenotypic variation. Defining how often Hsp90 occupies each of these roles is important but requires examining genetic variation that has not been exposed to natural selection. This is because Hsp90-potentiated phenotypic variation is visible to selection under normal conditions, whereas Hsp90-buffered phenotypic variation may not be (Fig 2). Thus, standing genetic variation might be biased to provide evidence for buffering (Fig 2).

In this issue of *PLOS Biology*, Geiler-Samerotte, Siegal, and coauthors measure the degree to which Hsp90 buffers and potentiates the effects of new mutations [15]. To do this, the authors utilize a panel of yeast mutation accumulation (MA) lines, which accrued mutations during many generations of growth in the absence of selection. They measure the heritable phenotypic variation shown by these MA lines—as well as by wild isolates and cross progeny from the same species—before and after disruption of Hsp90. They then compare how these three groups, which differ in their exposure to selection, respond to Hsp90 perturbation.

Following Hsp90 impairment, heritable phenotypic variation is reduced in the MA lines but increased in the isolates [15]. Given that the MA lines have not been exposed to selection and the isolates have, this result implies that Hsp90 predominantly acts as a potentiator and that selection has acted against Hsp90-potentiated phenotypic variation in nature. Consistent with this interpretation, cross progeny that carry new combinations of standing variants also show reduced heritable phenotypic variation upon Hsp90 perturbation [15]. This finding suggests that standing variants with Hsp90-potentiated effects are common in nature, but combinations of these variants that produce extreme phenotypes are selected against. Together, the results from the MA lines and cross progeny, both of which represent groups of strains that have not been biased by selection, support a view that Hsp90 acts more as a potentiator than a buffer.

## Hsp90 Is a Global Modifier That Shows Extensive Epistasis

**Fig 3:**
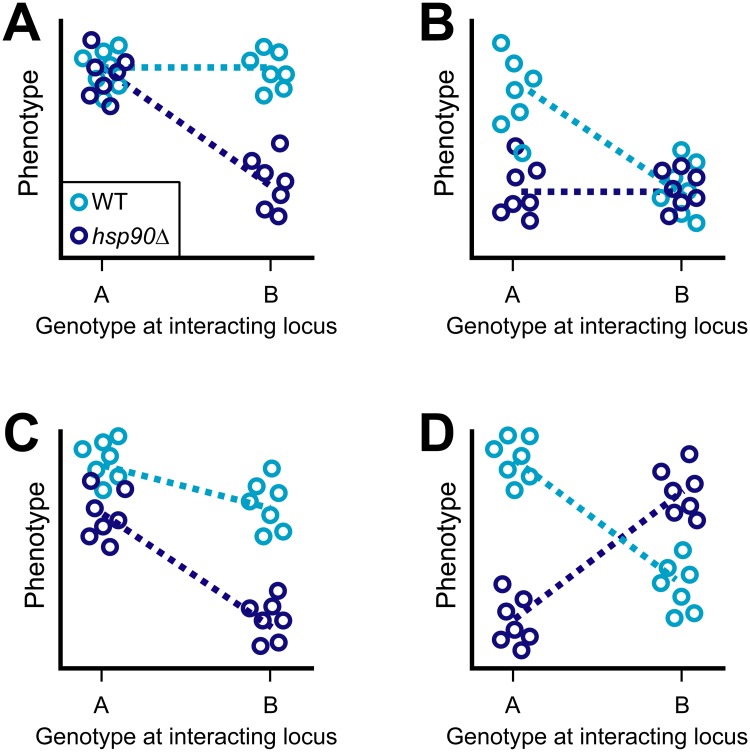
Hsp90 shows different forms of epistasis with individual genetic variants. Transformations of the genotype–phenotype map that occur following Hsp90 perturbation likely reflect the composite effect of multiple variants that show different types of epistatic interactions with Hsp90. In **A** and **B**, we illustrate how Hsp90 can buffer (**A**) or potentiate (**B**) the effects of individual variants. Furthermore, in **C** and **D**, we show how Hsp90 may also behave as a quantitative modifier that exhibits magnitude (**C**) or sign epistasis (**D**) with some variants. In each panel, the two alleles of a variant are shown along the *x*-axis, represented by “A” and “B.” On the *y*-axis, the phenotypes associated with these variants are shown when Hsp90 is functional and compromised using light and dark blue, respectively. Each circle represents a genetically distinct haploid. For a given Hsp90 state, lines indicate the difference in phenotype between individuals carrying the alternate alleles of the interacting variant.

With continued research, the utility of the terms buffer, capacitor, and potentiator becomes less clear. Not only do some of these words have meanings that seem mutually exclusive (e.g., buffer versus potentiator), but their relevance is also shaped by contextual factors, such as the type of genetic variation being examined and its exposure to selection. Moreover, these terms are often used to explain increases and decreases in heritable phenotype variation, which are in fact mediated by specific genetic variants that may show different types of responses to Hsp90 perturbation [3,15–17] (Fig 3).

In light of these considerations, the standard genetics concept of epistasis (or genetic interaction) may be a more straightforward way to communicate Hsp90’s effect on heritable phenotypic variation [15,18]. Epistasis occurs when the effect of one variant depends on one or more other variants [19–22]. Thus, variants that show changes in effect upon Hsp90 perturbation by definition epistatically interact with Hsp90.

Examining response to Hsp90 perturbation from the perspective of epistasis has multiple advantages. This framing encompasses situations in which Hsp90 buffers or potentiates the effects of variants (Fig 3). However, it also accommodates quantitative changes in the effect magnitude or sign of epistatically interacting variants (Fig 3). These quantitative epistatic interactions have received limited discussion in the Hsp90 literature but are prevalent in nature [23,24,42].

Epistasis can also be used to convey Hsp90’s global impact on the effects of genetic variants. Typically, a variant that influences the effect of another through an epistatic interaction is called a modifier. Therefore, we propose that Hsp90 be referred to as a global modifier because of the fact that it may show different types of epistatic interactions with many variants. The term global modifier is a more general way to describe Hsp90’s effect on the genotype–phenotype map than the buffer, capacitor, and potentiator concepts. Notably, this term remains applicable across the different contexts in which one might investigate response to Hsp90 perturbation.

## Questions Regarding Hsp90 and Other Global Modifiers

Research on Hsp90 has been instrumental in showing the significant influence that global modifiers can exert upon the genotype–phenotype map. Moving forward, it will be important to address multiple major questions in this area, including:

### Which Genes Behave as Global Modifiers?

Evidence suggests that Hsp90 is not unique in its ability to act as a global modifier. For example, a number of studies have shown that genes involved in chromatin regulation and transcriptional repression uncover large amounts of cryptic variation when perturbed [7,25–27] and that prions [28–30] and proteins with regions of intrinsic disorder [31] can behave in a similar manner to Hsp90. Moreover, many genes buffer the effects of environmental variation; these genes might also modify the effects of genetic variation [32]. Future screens will hopefully clarify the space of genes that are global modifiers.

### What Is the Genetic Architecture That Underlies Response to a Global Modifier’s Perturbation?

Much of the work on global modifiers to date has inferred alterations to the genotype–phenotype map from increases or decreases in heritable phenotypic variation. Yet, such changes do not provide direct insights into the number of genetic variants and forms of epistasis underlying these responses. This information can be obtained through statistically powerful genetic mapping experiments focused on standing genetic variation [23,33] or comprehensive analysis of MA lines that harbor known new mutations [15,26].

### What Are the Mechanisms by Which Global Modifiers Act?

Systematically resolving the exact variants that interact with particular global modifiers can shed light on how these genes influence heritable phenotypic variation. Epistatic interactions between a global modifier and variants may arise through a mixture of direct and indirect functional relationships. For instance, Hsp90 physically interacts with an array of client proteins, and amino acid changes in these clients might impact response to Hsp90 perturbation [13,14]. At the same time, epistatic interactions can involve variants in genes that have less direct functional relationships if these genes act in regulatory networks [7,34–36] or in compensatory or parallel cellular processes [37].

### To What Extent Do Global Modifiers Harbor Functional Variation?

Although global modifiers influence the effects of genetic variants in other genes, how much polymorphisms in global modifiers themselves contribute to heritable phenotypic variation remains unclear. For example, *cis* regulatory polymorphisms causing decreases in Hsp90 expression segregate in wild populations of *Drosophila* and alter the effects of other variants [38]. However, these Hsp90 polymorphisms are highly deleterious and quite rare, suggesting they are not a major source of heritable phenotypic variation. In contrast, the discoveries of variants that affect levels of phenotypic variability in mapping studies [39,40] or show epistatic interactions with many other variants [41] are consistent with the possibility that polymorphisms in global modifiers might contribute to heritable phenotypic variation in some populations.

## Conclusion

Hsp90 significantly influences the genotype–phenotype map through to its epistatic interactions with genetic variants on a global scale. For this reason, it is appropriate to describe Hsp90 and genes that behave similarly as global modifiers. Major questions to address in this area moving forward regard the broader space of genes that can act as global modifiers, the mechanisms by which Hsp90 and other global modifiers modulate the genotype–phenotype map, and the extent to which functional polymorphisms in global modifiers themselves affect heritable phenotypic variation. Research on these topics should improve our understanding of the relationship between genotype and phenotype.
